# Real-World Data and Budget Impact Analysis (BIA): Evaluation of a Targeted Next-Generation Sequencing Diagnostic Approach in Two Orthopedic Rare Diseases

**DOI:** 10.3389/fphar.2022.785705

**Published:** 2022-06-06

**Authors:** Elena Pedrini, Antonella Negro, Eugenio Di Brino, Valentina Pecoraro, Camilla Sculco, Elisabetta Abelli, Maria Gnoli, Armando Magrelli, Luca Sangiorgi, Americo Cicchetti

**Affiliations:** ^1^ Department of Rare Skeletal Disorders, IRCCS Istituto Ortopedico Rizzoli, Bologna, Italy; ^2^ Regional Agency for Health and Social Care of Emilia-Romagna, Bologna, Italy; ^3^ Università Cattolica del Sacro Cuore, Graduate School of Health Economics and Management, Rome, Italy; ^4^ School of Economics and Management, Alma Mater Studiorum-University of Bologna, Bologna, Italy; ^5^ National Center for Drug Research and Evaluation, National Institute of Health (ISS), Rome, Italy

**Keywords:** Budget Impact Analysis (BIA), NGS, multiple osteochondromas, osteogenesis imperfecta, rare diseases

## Abstract

**Objective:** Next-generation sequencing (NGS) technology, changing the diagnostic approach, has become essential in clinical settings, and its adoption by public health laboratories is now the practice. Despite this, as technological innovations, its intake requires an evaluation of both the clinical utility and the economic investment, especially considering the rare disease scenario. This study evaluated the analytical validity and the budget impact of an NGS-Ion Torrent™ approach for the molecular germline diagnosis of two musculoskeletal rare diseases.

**Methods:** Two cohorts of 200 and 199 patients with suspect or clinical diagnosis of multiple osteochondromas (MO) and osteogenesis imperfecta (OI) previously evaluated with a single-gene diagnostic protocol were re-analyzed using a targeted NGS assay. Analytical validity was assessed by comparing NGS and single-gene protocol. A budget impact analysis using real-world cost data-considering the healthcare perspective— was performed by applying activity-based costing (ABC). The cost considered consumables, personnel, and equipment. Additional costs not related to NGS activities were not considered. Sensitivity analysis was performed.

**Results:** The NGS method showed a higher (for MO) and comparable (for OI) diagnostic sensitivity than the traditional techniques, apart from always reducing the time and costs of diagnosis. Overall, the cost saving per patient is € 765 for OI and € 74 for MO. Materials represented the highest cost driver of the NGS process. A time saving—proportional to the panel size—has been assessed in both cases.

**Conclusions:** Our targeted NGS diagnostic approach decreases time to diagnosis and costs, appearing to be beneficial and recommended both for patients and from a healthcare perspective in routine diagnosis also considering very small gene panels and a low patient flow. The adequate analytical sensitivity always required the additional Sanger sequencing step of the low- and non-covered regions. A more accurate strategy evaluation is suggested in the case of ultra-rare/complex diseases, large gene-panel, or non-reference diagnostic centers.

## Introduction

Sanger sequencing has always been considered the gold standard approach to detect genetic alterations responsible for several human diseases. To speed up genetic tests, several pre-screening methods such as denaturing high-performance liquid chromatography (DHPLC) and high-resolution melting (HRM) have been added over time, up to the coming out of the massively parallel sequencing technology known as next-generation sequencing (NGS) which can analyze simultaneously a large number of genes. This new method drastically changed the genetic diagnostic approach, replacing traditional single-gene molecular diagnostic tests and becoming essential for the numerous genetically heterogeneous disorders. Due to the several applications in clinical settings (i.e., personalized precision medicine, diagnosis, and pharmacogenomics), adoption of NGS by public health laboratories is now the practice, with a peak of platform acquisition in the last few years; despite this, as technological innovations represent a major cost driver in health care (Bodenheimer, 2005), its intake requires an evaluation of both the clinical utility and the economic investment. To assess this issue, a multidimensional analysis such as the health technology assessment (HTA) has gained importance over the last few years. In this context, the budget impact analysis (BIA) is an element to support and develop a multidisciplinary process that uses explicit methods to determine the value of the health technology at different points in its lifecycle. The purpose is to inform the decision-making process to promote an equitable, efficient, and high-quality health system (O'Rourke et al., 2020). When making changes to an already available diagnostic protocol, it is essential to evaluate not only the economic aspects but also the effectiveness of the new technology which must be as efficient as the “current” detection methods (McCourt et al., 2013). This is especially true when considering the limitations of the NGS techniques in the presence of GC-rich sequences or homopolymer regions which require an accurate preliminary technical validation.

NGS-based genetic testing comprises different options (i.e., targeted screening, whole-exome sequencing, and whole-genome sequencing) and different technologies whose utility usually depends on the purpose and clinical condition (Goodwin et al., 2016). According to our rare diseases’ diagnostic context, the choice fell on the acquisition of the Ion Torrent PGM technology (Thermo Fisher Scientific, US) whose easily adjustable throughput range (30 Mb–2 Gb) and runtimes (between 2 and 7 h) make it faster than other dominating platforms and flexible enough for the management of small gene panels with a limited and variable number of patients.

Few published studies are currently available on the reliability and cost-effectiveness of NGS, especially focused on the Illumina technology and oncologic diseases (Hagemann et al., 2015; Doble, 2016; Goodwin et al., 2016; Joosten et al., 2016; Marino et al., 2018), whose specific technical features and analytical needs make results not applicable to all clinical fields. Two previous studies demonstrated the usefulness of targeted NGS in molecular diagnosis using the Ion Torrent technology (Stoddard et al., 2014; Al-Mousa et al., 2016); despite this, the cost evaluation was limited to the consumables and the use of big panel sizes (173 and 162 genes, respectively) was in line with the already known advantages of the NGS technology in the presence of a large number of samples and genes. Focusing our attention on a different scenario, in terms of patients’ number, NGS platforms, gene panel size, and mutation types (germline and not somatic), we considered the use of the Ion Torrent NGS platform in the molecular diagnosis of two skeletal rare diseases characterized by different levels of genetic heterogeneity and clinical complexity: multiple osteochondromas (MO) and osteogenesis imperfecta (OI). Multiple osteochondromas (OMIM#133700) is an autosomal dominant cartilaginous disease (incidence: 1/50,000) with an easy clinical diagnosis and caused by germline mutations in *EXT1* and *EXT2* genes (Pacifici, 2017). Differently, osteogenesis imperfecta (OMIM#166200, 166210, 259420, and 166220) is a phenotypically and genetically heterogeneous disease of the connective tissue (incidence: 1/20,000) with both dominant and recessive transmission (Marini et al., 2007) related to an ever-increasing number of genes including *COL1A1*, *COL1A2, IFITM5*, *CRTAP*, *LEPRE1*, *PPIB*, *SERPINF1*, *WNT1*, *TMEM38B*, *BMP1*, *SERPINH1*, *FKBP10*, *SP7*, and *PLS3*. Although the clinical features and the family history of OI patients usually guide the selection of genes to be tested, the wide phenotypic heterogeneity, ranging from nearly asymptomatic individuals with occasional fractures to neonatal lethality, the presence of atypical cases, and the deficiency of medical history data could result in laborious and sometimes inefficient molecular screening procedures.

The genetic testing approach offered by Istituto Ortopedico Rizzoli (IOR, Bologna, Italy) in the molecular diagnosis of rare diseases was traditionally based on long and expensive single-gene analyses carried out through multi-step protocols including Sanger sequencing, DHPLC, and MLPA techniques. The acquisition of the Ion Torrent PGM system has made possible the use of NGS panels to simultaneously test all disease-related genes and speed up the diagnostic process, an essential issue for rare patients. Indeed, even if treatments for rare disorders are often limited, a definitive and quick diagnosis saves patients from unnecessary additional tests and clinical examinations, improving both their assistance—often poor—and those of their families (Lee et al., 2010; Carmichael et al., 2015). This is why the use of the NGS system in our specific diagnostic field represents the best available option.

In this study, we evaluated the effectiveness and the budget impact of a targeted NGS-Ion Torrent™–based assay in substitution of the traditional single-gene protocols to have sensitive and accurate diagnostic tools for MO and OI patients. We considered the healthcare perspective and the real scenario (without any cost assumption) in a rare diseases’ diagnostic context related to the use of very small (12.78 and 110.89 kb) NGS panels with a limited number of patients.

## Materials and Methods

### Patient Population

200 MO and 199 OI patients visited at the Department of Rare Skeletal Disorders (IRCCS Istituto Ortopedico Rizzoli, Bologna, Italy) with suspect or clinical diagnosis of disease were included in the study. All the patients were previously evaluated for the presence of pathogenic mutations using a “single-gene protocol” based on the use of denaturing high-performance liquid chromatography (DHPLC), multiplex ligation–dependent probe amplification (MLPA), and Sanger sequencing, depending on the gene. 182 MO and 148 OI patients had an already known genetic diagnosis detected using “single-gene” tests: 101 MO and 111 OI patients had single nucleotide substitutions, 65 MO and 35 OI patients had small insertions/deletions (indels), and 16 MO and 2 OI patients had big deletions. The remaining patients were classified as undiagnosed MO and OI patients. All the enrolled patients were re-evaluated by NGS. For each patient, the genomic DNA—when not already available—was isolated from peripheral blood or buccal swab using an automated workstation (Biomek NX with Agencourt Genfind v2 DNA Isolation Tube Kit Beckman Coulter) or a manual kit (Oragene-DNA, Voden Medical), respectively. For each DNA extraction, we obtained 3 μg from peripheral blood and 9 μg from the buccal swab. This project was approved by the Ethics Committee of Istituto Ortopedico Rizzoli (20-12-2017/No. 0012819). All research works were performed in accordance with relevant guidelines/regulations. The informed consent was obtained from all participants and/or their legal guardians.

### Genetic Analysis Workflows

Our “traditional protocols” consisted in testing one gene at a time; the disease-related genes were sequentially analyzed depending on clinical features and known mutation frequencies, as described in Supplementary Figures 1–2. When requested by geneticists, only a few specific genes were included in the workflow. For each gene, all coding regions and intron–exon junctions were considered. Multiple osteochondromas diagnosis was performed using a multi-step approach where probands were first tested for *EXT1* (11 exons/16 amplicons)—which contains most of the MO-related variants—and then for *EXT2* (14 exons/14 amplicons). Both genes were analyzed with a pre-screening test performed by DHPLC (3500 HT Wave Nucleic Acid Fragment Analysis System, Transgenomic Inc.) followed by Sanger sequencing (ABI PRISM 3500XL Genetic Analyzer, Thermo Fisher Scientific) of all abnormal profiles, as previously described (Jennes et al., 2008). In case of negative results, that is, absence of point mutations, MLPA screening (kit SALSA MLPA probemix, MRC-Holland) was performed to detect large deletions/duplications that account for about 5–6% of cases (Jennes et al., 2008). Osteogenesis imperfecta molecular diagnosis was performed by analyzing all the genes associated with a specific phenotype—when defined—following the order described in Supplementary Figure 2, unless otherwise requested. *COL1A1* (52 exons/43 amplicons) and *COL1A2* (52 exons/48 amplicons) were analyzed using a multi-step screening protocol by DHPLC, followed by direct sequencing of all abnormal elution profiles (Gentile et al., 2012). In case of the absence of pathogenic point mutations, the detection of big deletions/duplications—accounting for about 1–2% of cases—was performed using MLPA. All the other genes associated with OI were analyzed by pure Sanger sequencing (*IFITM5:* 2 exons, *CRTAP:* 7 exons, *LEPRE1:* 15 exons, *PPIB:* 5 exons, *SERPINF1:* 8 exons, *WNT1:* 4 exons, *TMEM38B:* 6 exons, *BMP1:* 20 exons, *SERPINH1:* 5 exons, *FKBP10:* 10 exons, *SP7:* 3 exons, and *PLS3:* 16 exons) for a total of 104 Sanger fragments.

All Sanger sequencing data were aligned against the reference sequences and evaluated for the presence of variants by the commercial SEQPATIENT application (JSI medical systems GmbH, Germany).

The “NGS protocol” is based on the use of the Ion Torrent PGM platform (Thermo Fisher Scientific, Waltham, MA, United States) to analyze simultaneously all genes related to the diseases. Two custom panels—were created by Ion AmpliSeq Designer software (Thermo Fisher Scientific) as follows: a 12.78 kb panel (2 primer pools) was set up to cover both coding and UTR regions of *EXT1* and *EXT2* with at least 50-bp flanking intronic regions, whereas a 110.89 kb panel (2 primer pools) was designed to cover all coding regions of the previously described OI-related genes, including at least 25-bp flanking intronic regions. Primer designs generate 125–275 bp and 125–375 bp amplicons, respectively, providing 99.37% and 98.11% coverage of the selected regions. Due to the low number of genes, the panel design could not be optimized for CNV detection using the Ion Reporter Software, the tool supporting the Ion Torrent data analyses. Ten nanograms of DNA per sample were processed using the Ion AmpliSeq^TM^ Library Kit 2.0 (Thermo Fisher Scientific). To further speed up the analysis, the Ion Chef platform (Thermo Fisher Scientific) was used to automate the chip loading, the Ion 314 Chip Kit v2 BC was used for MO samples, and the Ion 316 Chip Kit v2 BC for OI samples. To obtain a mean coverage depth of 300X and minimize the per-sample sequencing cost, 20 MO samples were loaded on Ion 314 Chip and 16 OI samples on Ion 316 Chip; considering our average annual flow of patients, this loading amount translates into a minimum of three NGS runs per year for each pathology. Data analyses were performed using commercial applications: the Ion Reporter^TM^ Software (Thermo Fisher Scientific) and the SEQNext application (JSI medical systems GmbH, Germany). All suspected pathogenic variants were confirmed by Sanger sequencing. In case of negative results, all regions not covered or with a low read depth (<30X) were analyzed by Sanger sequencing. In the absence of point mutations, big deletions/duplications were investigated as follows: in the case of MO samples, CNV detection was performed using a specific algorithm based on the average amplicon coverage normalization, previously validated on 10 MO samples known to carry a multi-exon deletion; all detected CNVs were confirmed by quantitative real-time PCR. In the case of non-suitability of the coverage data, CNV detection was performed using MLPA. In the case of OI diagnosis, the absence of enough positive reference samples did not allow us to validate the same method for CNV detections in *COL1A1* and *COL1A2*; big deletion/duplication detection was, therefore, performed by MLPA, as in the traditional protocol.

### Analytical Validity

Analytical validity describes the accuracy of the test in detecting the mutation of interest. To evaluate the technical performance of the Ion Torrent NGS platform, we considered the concordance of the results obtained with the two diagnostic protocols (traditional and NGS-based). True-positive (TP) and false-negative (FN) variant calls were defined to calculate the sensitivity as follows: TP/(TP + FN); true positives (TPs) were variants detected by the diagnostic protocol and present in the patients whereas false negatives (FNs) were variants not recognized by the procedure. To evaluate the appropriateness of the inclusion of the Sanger sequencing step to examine all regions with no or low reading depth, whose addition increases the costs, we estimated the diagnostic sensitivity also considering the pure NGS analysis.

### Hands-On Time for Diagnostic Procedures

Time estimation was performed for wet laboratory procedures and data analysis to estimate the efficiency of the new NGS diagnostic protocol, besides being essential to define the personnel costs, as described below. The hands-on time required for all molecular diagnostic steps (i.e., DNA extraction, DHPLC, MLPA, real-time PCR, Sanger sequencing, NGS, Sanger sequencing data analysis, and NGS data analysis) was calculated considering the time for the manual procedures, even if part of an automated protocol. Hands-on times for each molecular procedure comprise sample preparation and run set-up; raw data processing and data interpretation were included in the time for data analysis. Time-per-sample and time-per-amplicon were calculated considering our higher optimized sample-size workflow obtaining the hands-on times for each diagnostic step as detailed in Table 1. The time workload reported in our study for the different molecular techniques is in line with what was previously described in Italian molecular laboratories (Tagliafico et al., 2018), except for the NGS data analyses; our data showed a reduced time for NGS data analysis, probably related to the easy-to-use commercial software for variant detections and the high percentage of patients carrying a pathogenic variant.

**TABLE 1:** Hands-on time for each molecular diagnostic step considering an average.

Activity	Hands-on times (min)
DNA extraction from blood	7 (per sample)
DNA extraction (from buccal swab)	15 (per sample)
DHPLC screening (EXT1)	22.86 (per sample)
DHPLC data analysis (EXT1)	8.6 (per sample)
DHPLC screening (EXT2)	21.4 (per sample)
DHPLC data analysis (EXT2)	8.6 (per sample)
DHPLC screening (COL1A1)	59.8 (per sample)
DHPLC data analysis (COL1A1)	22.4 (per sample)
DHPLC screening (COL1A2)	67 (per sample)
DHPLC data analysis (COL1A2)	25.1 (per sample)
MLPA (per gene)	6.4 (per sample)
MLPA data analysis (per gene)	3.5 (per sample)
Sanger sequencing	5 (per amplicon)
Sanger sequencing data analysis	2.5 (per amplicon)
Real-time PCR	9 (per amplicon)
Real-time PCR data analysis	3 (per amplicon)
NGS (multiple osteochondromas, 2 genes)	37.5 (per sample)
NGS data analysis (multiple osteochondromas, 2 genes)	10 (per sample)
NGS (osteogenesis imperfecta, 14 genes)	25 (per sample)
NGS data analysis (osteogenesis imperfecta, 14 genes)	50 (per sample)

DHPLC, denaturing high-performance liquid chromatography; MLPA: multiplex ligation–dependent probe amplification.

### Budget Impact Analysis

To carry out the economic assessment, a budget impact model (BIM) (Mauskopf et al., 2007) was developed by ALTEMS (Graduate School of Health Economics and Management, Università Cattolica del Sacro Cuore, Roma, Italy), considering the healthcare perspective. This model is commonly used by decision makers for planning a new intervention adoption by estimating the financial consequences. The analysis applied the typical methods of activity-based costing (ABC, Budget Impact Analysis, 2016) to identify the cost drivers associated with each type of diagnostic protocol (single gene–based and NGS-based). To determine the impact in terms of absorbed resources associated with the introduction of the NGS device, a BIM was implemented based on the comparison of two alternative scenarios in the Italian healthcare context:

– a scenario based on a single-gene diagnostic protocol (as is);
– an alternative scenario based on the use of NGS (to be).

The costs were calculated considering the process from DNA extraction, library preparation, sequencing, and bioinformatic analysis; other costs related to receiving the blood sample, drafting the clinical reports, providing an independent evaluation by a second professional, issuing the final clinical report, and storing the data have not been considered since—even if costly—they do not change in the two molecular approaches. The analyses are based on the total samples and the results derived from the volume of activity provided. The ABC technique allows calculating the full cost of a product/service by measuring the cost of each activity/resource connected to it. The costs related to the sample are not attributed to the total sample number but directly to the activities that generate them and are understood as the actual determinants of the cost. ABC analysis was divided into three distinct phases:

1) Identification of resources: all resources required for each step of both molecular methods have been identified (equipment, consumables, and personnel), thereby allowing the determination of the professionals who actively intervene, including the time spent on each activity.
2) Cost measurement: for each healthcare professional, the average cost divided by category was calculated and the average expenditure for the materials was used.
3) Valorization of results: monetary values have been attributed to the respective cost drivers.

Equipment costs were calculated considering both usage and maintenance. We used the acquisition price at the time of the study without discount, linearly amortized considering a 5-year period, and then adjusted according to the utilization. Machine usury was not considered. Maintenance costs were calculated using the annual full-risk contract fees and then adjusted, according to each diagnostic device’s utilization. Supplementary Table 1 can be referred for a detailed description of the input data considered for the diagnostic devices. Personnel costs were evaluated, considering the time spent and the role. A technician is involved in all manual procedures for the sample preparation, whereas a biologist/bioinformatician is required for all analytical activities. We assumed, according to the Italian Legislation, that the annual working times for personnel were 36 h a week. The costs per hour considered for each healthcare professional are detailed in Supplementary Table 1. The hands-on time for each diagnostic step is detailed in Table 1, as previously described. The cost of labor per patient (for each diagnostic procedure) was calculated from the time taken to provide each activity and the real number of activities performed. The consumable costs were calculated according to our real consumption. The time horizon of the analysis is one year. The unit costs were considered without discounts.

Results are presented as total cost per patient (cost per sample). The costs are expressed in euros. All statistical analyses were performed using Microsoft Excel 2000. The model includes the one-way sensitivity analysis for determining the uncertainty characterizing the parameters considered in the model, thus identifying the drivers whose deviation has a significant impact on the results obtained in the base case. In the sensitivity analysis, parameters’ values in the 25^th^ and 75^th^ percentile of the normal inverse distribution were calculated and represented in the Tornado chart, showing the differential cost resulting from the base-case analysis as the central value (Briggs et al., 2012).

## Results

### Diagnostic Efficiency: Multiple Osteochondromas

The “traditional protocol” (DHPLC–Sanger–MLPA) detected 182 pathogenic variants (101 SNVs, 65 small indels, and 16 big deletions) in as many patients (91%): 136 in *EXT1* and 46 in *EXT2*.

The new “NGS protocol” (NGS–Sanger) identified 184 (92%) causative variants (102 SNVs, 65 small indels, and 17 big deletions), 137 in *EXT1* and 47 in *EXT2*. The new finding mutations were a missense variant (NM_000127.2: c.1397C>G, p.Pro466Arg) in *EXT1* and a big deletion comprising a part of exon 5 of *EXT2*, a region not covered by MLPA probes. No false-positive variants were detected, thereby not requiring additional Sanger sequencing to remove these calls. Nevertheless, in addition to 163 Sanger sequencing analyses performed to confirm NGS-detected variants, 18 cases (9%) required the Sanger approach to analyze some low read depth regions; this additional step allowed the identification of four indel variants not detected by the pure NGS assay. All analytical steps required for both diagnostic approaches are detailed in Supplementary Table 2A.

Considering all 184 MO-related variants detected using both assays, the traditional protocol was able to correctly detect mutations (SNVs, small indels, and CNVs) in 182 of 184 samples, with an overall sensitivity of 98.9%. The pure NGS analysis—without the Sanger sequencing of <30X-coverage regions—revealed the presence of the pathogenic variant in only 180 samples, increasing the false-negative rate (2,2%, four false-negative results) and reducing the sensitivity (97.30%). When adding the Sanger sequencing step to examine all low read depth regions, the overall sensitivity achieved 100% (Table 2A). Decomposing the sensitivity by the mutation type, the sensitivity for variant detection using the traditional protocol was 99% (101/102) for detecting SNVs, 100% (65/65) for detecting small indels, and 94% (16/17) for detecting CNVs with MLPA. Considering the pure NGS assay, the sensitivity for detecting SNVs was 100% (102/102), that for detecting small indels was 93.8% (61/65), and that for detecting CNVs was 100% (17/17). Considering the NGS assay with the additional Sanger sequencing step, the sensitivity of detection for all the mutation types was 100%.

**TABLE 2:** Comparisons among the number of true-positive pathogenic variants, false-negative (missed) calls, and sensitivity associated with the traditional protocol, the NGS pure assay, and the NGS assay with Sanger sequencing of all low read depth regions. In brackets, the same values are divided by the mutation type.

A	Multiple osteochondromas		
	**Traditional protocol (SNVs, small indels, and CNVs)**	**Pure targeted NGS assay (SNVs, small indels, and CNVs)**	**NGS protocol targeted NGS assay + Sanger sequencing of <30X coverage regions (SNVs, small indels, and CNVs)**
True positives	182 (101, 65, and 16)	180 (102, 61, and 17)	184 (102, 65, and 17)
False negatives	2 (1, 0, and 1)	4 (0, 4, and 0)	0
Sensitivity	98.9% (99%, 100%, and 94%)	97.8% (100%, 93.8%, and 100%)	100% (100%, 100%, and 100%)
**B**	**Osteogenesis imperfecta**		
	**Traditional protocol (SNVs, small indels, and CNVs)**	**Pure targeted NGS assay (SNVs, small indels, and CNVs)**	**NGS protocol targeted NGS assay + Sanger sequencing of <30X coverage regions (SNVs, small indels, and CNVs)**
True positives	148 (112, 34, and 2)	142 (109, 31, and 2)	148 (112, 34, and 2)
False negatives	0	6(3,3, and 0)	0
Sensitivity	100% (100%, 100%, and 100%)	95.9% (97.3%, 91.2%, and 100%)	100% (100%, 100%, and 100%)

SNVs, single-nucleotide variants; CNVs, copy number variations.

### Diagnostic Efficiency: Osteogenesis Imperfecta

The “traditional protocol” revealed a mutation associated with OI in 148/199 (74.4%) patients (112 SNVs, 34 small indels, and 2 big deletions): 64 in *COL1A1*, 68 in *COL1A2*, 3 in *CRTAP*, 1 in *FKBP10*, 3 in *IFITM5*, 3 in *LEPRE1*, 1 in *PLS3*, 3 in *SERPINF1*, and 2 in *WNT1*.

The “NGS protocol” confirmed all the previously detected variants. An average of 1.6 false positives per patient were revealed—all represented by small indel variant calls—which require confirmation by Sanger sequencing in the absence of a clear pathogenic variant; to remove these calls, 84 additional Sanger sequencing analyses were performed. Overall, 53 cases (26.6%) negative to the presence of pathogenic variants required the Sanger sequencing analysis of all the false-positive calls and the low NGS read depth regions, and 331 sequences were performed allowing the detection of six variants missed using the pure NGS assay. All analytical steps required for both diagnostic protocols are detailed in Supplementary Table 2B.

Considering the diagnostic reliability, the new NGS protocol has sensitivity as high as the traditional protocol (100%). Of note, if not associated with the additional Sanger sequencing step in case of negative results, the diagnostic sensitivity of the pure NGS assay drops significantly, reaching 95.9% (Table 2B).

### Timing of “Targeted NGS Protocol” Versus “Traditional Protocol”

Considering the time estimation of laboratory procedures to complete a molecular diagnosis with targeted NGS and traditional single-gene protocol, we assessed the time-saving attribute in both diseases when using the NGS approach; assuming that 100% is the time per sample required to complete the genetic screening with the traditional protocol, the mean time with the NGS-based procedure is 87.8% for MO molecular testing and 34.9% for the OI one, making the time-saving feature proportional to the panel size. Comparing the absolute average time (per patient) required for the sample preparation and the data analysis in the NGS protocols, it is 47.3 and 14.2 min in the MO genetic test and 48.6 and 59 min in the OI, respectively, pointing out a comparable manual activity and an increase in data analysis time proportional to the panel size. Of note, the extremely short time for data interpretation in the MO genetic test is due to highly favorable conditions represented by the very small gene panel, the use of a user-friendly application for NGS data analysis, and the high percentage of MO patients (90%) with a positive molecular diagnosis.

### Activity-Based Costing Results

We compared the costs of all procedures from DNA extraction to the pathogenic variant detection for both the targeted NGS methods and our traditional single-gene diagnostic method. Aggregating the costs of material, personnel, and sequencing device, we have the base case represented in Table 3. Considering MO molecular testing, the total cost per patient is € 388 with the traditional protocol (equipment: € 75, personnel: € 33, and consumables: € 280) and € 314 with the NGS-based procedure (equipment: € 41, personnel: € 29, and consumables: € 244), with a mean saving of € 74 (−19%). Although the greatest cost reduction is related to the equipment (−45%), the material costs remain the most impacting overall (49%), followed by the equipment (46%).

**TABLE 3:** Cost analysis, single-gene protocol versus NGS protocol.

Multiple osteochondromas—total costs (cost per patient)					
Cost	Traditional protocol	NGS protocol	Differential cost	D differential cost (%)	Weight difference (%)
Equipment	€ 15,097.4 (€ 75)	€ 8,257.7 (€ 41)	−€ 6,821.7 (-€ 34)	−45%	46%
Personnel	€ 6,522.8 (€ 33)	€ 5,757.8 (€ 29)	−€ 765 (-€ 4)	−12%	5%
Material	€ 56,018.2 (€ 280)	€ 48,827.0 (€ 244)	−€ 7,191.2 (-€ 36)	−13%	49%
Total	€ 77,638.3 (€ 388)	€ 62,860.5 (€ 314)	−€ 14,777.9 (-€ 74)	−19%	100%
**Osteogenesis imperfecta—total costs (cost per patient)**					
**Cost**	**Traditional protocol**	**NGS protocol**	**Differential cost**	**D differential cost (%)**	**Weight difference (%)**
Equipment	€ 33,224.2 (€ 167)	€ 14,099.2 (€ 71)	−€ 19,125 (−€ 96)	−57%	13%
Personnel	€ 26,611.7 (€ 134)	€ 11,294.1 (€ 57)	−€ 15,317.6 (−€ 77)	−57%	10%
Material	€ 196,380.0 (€ 982)	€ 78,075.5 (€ 390)	−€ 118,304.5 (−€ 592)	−60%	77%
Total	€ 256,215.9 (€ 1,283)	€ 103,468.8 (€ 518)	−€ 152,747.1 (−€ 765)	−60%	100%

NGS, next-generation sequencing; traditional protocol: single-gene test.

Analyzing OI genetic screening, the total cost per patient is € 1283 with the traditional protocol (equipment: € 167, personnel: € 134, and consumables: € 982) and € 518 with the targeted NGS assay (equipment: € 71, personnel: € 57, and consumables: € 390). Switching from the single-gene procedures to the NGS technology, we assessed a cost-saving per patient of € 765. Compared to MO genetic screening, the material and personnel cost savings are significantly higher (−60% and −57%), making them proportional to the number of genes included in the screening.

Focusing on the NGS assay and analyzing the consumable costs—related to the greatest absorption of resources—the mean cost is € 244 (range: € 205–306) for MO diagnosis and € 390 (range: € 322–616) for OI molecular screening, revealing an increase in the delta as the target size (in kB) increases. Since the higher costs are associated with cases in which a pathological variant is not detected—requiring additional tests to examine all not covered regions—a substantial increase in the mean cost is expected in the event of inappropriate clinical diagnoses, as well as with the increase of the panel size. Alternatively, in the case of NGS panels with the same target size, diseases characterized by a lower percentage of patients usually carrying a pathogenic variant will have higher diagnostic costs.

Finally, to estimate the cost impact of the complemented Sanger sequencing for filling the low coverage regions and verifying the false-positive calls, we calculated the ‘consumables and personnel’ costs related to this additional step. Considering the MO diagnostic assay, 19 patients required the Sanger sequencing step due to the absence of pathogenic variant calls with the pure NGS assay; the mean additional cost per patient for this activity is € 52.9 (range: € 18.3–54.9). Because 90% of MO patients are usually positive to the presence of *EXT1–EXT2* mutations, few patients require the additional step, thus making its impact not relevant; considering the mean total costs per patient, 1.8% is attributable to additional Sanger sequencing activities. Evaluating the OI diagnostic assay, 55 patients underwent the additional Sanger sequencing step with a related mean cost per patient of € 124.8 (range: € 36.6–292.6), considerably higher than that of the previous case; this is due to the higher number of genes (14 versus 2), the longer region length, and the not always clear clinical diagnosis, leading to a large number of patients negative to molecular screening (25.5%). Considering the whole OI case study, 7.5% of the average costs per patient are required to complete uncovered coding regions. The more correctly the clinical diagnosis is established, the less the economic impact of this additional step will be, making it essential to carefully evaluate the “diagnostic question”. At the same time, the higher the number of patients carrying a pathogenic mutation (e.g., 90% for MO patients), the lower the percentage of those that will require Sanger sequencing, making this additional step disease-related.

It must be underlined that our cost analysis does not represent the whole diagnostic workflow but only costs directly linked to genetic screening activities. Moreover, we did not consider other additional costs (overhead, quality costs, etc.), estimated to account for around 30% of the total costs (Marino et al., 2018).

### One-Way Sensitivity Analysis

**FIGURE 1:**
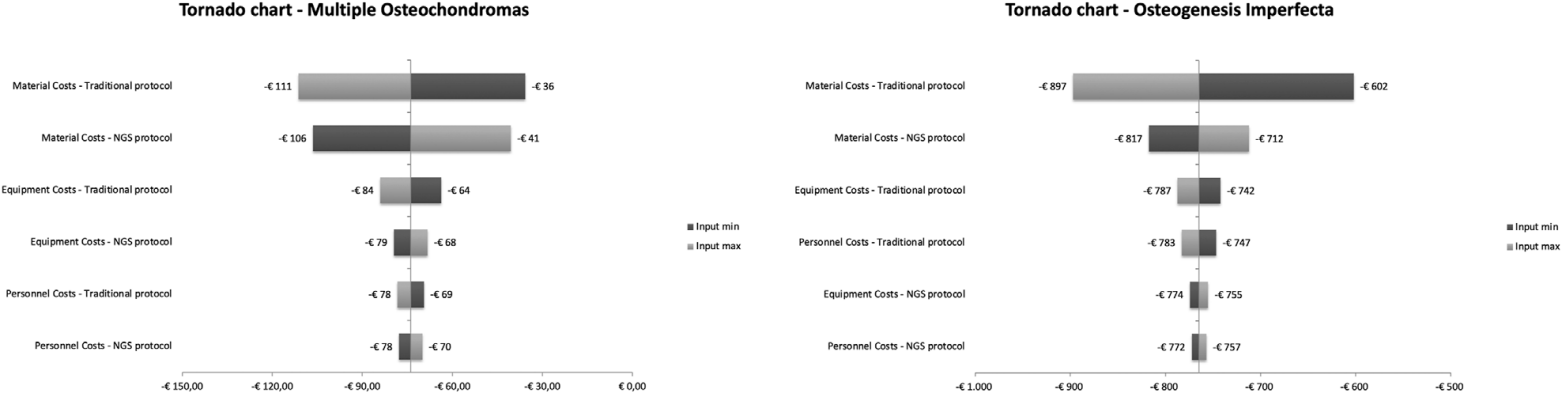
One-way sensitivity analyses to evaluate changes in savings of total costs.

To explore parameters’ uncertainty as compared to the base-case scenario, as well as to validate its robustness, a one-way sensitivity analysis was conducted. It has been assumed that a standard deviation of 25% is associated with each parameter included in the budget impact model. The results are presented in Figure 1. As previously highlighted (Marino et al., 2018), the parameter whose variation determines the greatest impact on base case’s results in both diagnostic protocols is related to the “material costs;” in this perspective, an increase in the consumable costs related to NGS activities would increase the total NGS cost, leading to a reduction in the savings determined in the base-case.

Conversely, “equipment” and “personnel”—with a limited impact on the total NGS costs—are characterized by less uncertainty and by a smaller impact in case of variation.

## Discussion

Even if a multidimensional analysis is required for the introduction of any innovative technology in health care (Bodenheimer, 2005; O'Rourke et al., 2020; Hutton et al., 2007), few studies—particularly oriented to Illumina technology and oncologic diseases—have been published on the use of NGS in medical genetics despite its widespread use. Moreover, most of the studies evaluated the economic impact of molecular activities only by considering the consumable costs (Stoddard et al., 2014; Al-Mousa et al., 2016). To fill this gap, the study proposes an effectiveness evaluation and a budget-impact assessment regarding the use of the Ion Torrent technology—a mid-length read, low cost, and high-speed NGS platform—in the substitution of the traditional single-gene testing in the molecular diagnosis of two rare skeletal diseases: multiple osteochondromas (MO) and osteogenesis imperfecta (OI). Despite the amplicon-based targeted-NGS approach being preferred in the case of small gene panels (Xue et al., 2015; Tagliafico et al., 2018) and the low throughput and flexibility of the Ion Torrent PGM technology being suitable to optimize a low patient flow, its efficiency may not be certain for an efficient and timely molecular analysis for diseases with a very low genetic heterogeneity (i.e., multiple osteochondromas). Our results indicate a higher (in MO disease) and equal (in OI disease) analytical validity for the NGS assays if compared to the traditional protocol, thus confirming their diagnostic efficiency for mutation detection. As an added value, the NGS method allowed to reach a molecular diagnosis in two MO patients who lacked a genetic diagnosis using the traditional protocol. Even though it was not the main purpose of the study, an accurate preliminary technical validation was essential to hypothesize the introduction of the new diagnostic tools in clinical practice. It is important to outline that we reached an adequate analytical sensitivity—meaning not lower than that of the traditional protocol—only with the additional Sanger sequencing step for filling in low- and no-coverage exons. When this additional step is required (i.e., whenever uncovered regions contain known mutational sites), a cost increase must always be considered; in our experience, we revealed a mean increase of € 52.9 and € 124.8 (respectively related to 2 and 14 target genes) in patients undergoing the additional step-related only to the costs considered in the study-making it proportional to the genetic heterogeneity and the complexity of the target regions. Since this additional step is carried out only in the absence of the pathogenic variant detected with the pure NGS assay, a substantial increase of the costs is expected—with the same target size—in the presence of frequent not-accurate or difficult clinical diagnoses, or when diagnosing diseases characterized by low mutation frequencies, both responsible for a high number of NGS negative patients. This means that the NGS costs are not purely related to the target region size unless limiting molecular screening to the well-covered regions (>30X), however, reducing the diagnostic confidence of the assay.

Particular attention should be paid to the molecular diagnosis of diseases with a high frequency of big deletions/duplications. Due to the MLPA technique limitations, the use of an NGS approach—with a validated CNV detection assay—is always preferable to improve the mutation detection rate as it examines the exonic and intronic regions not covered by the MLPA probes. In our study, we were able to efficiently detect heterozygous deletions in MO patients using a specific algorithm based on the average amplicon coverage normalization, thus overcoming the need for further MLPA analyses, resulting in time- and cost-saving. Using the new method, we detected a previously undetected pathogenic large deletion, thereby increasing the diagnostic detection rate in MO disease. Due to the lack of an adequate number of positive controls, we could not validate the same method for OI molecular screening, thus requiring the traditional MLPA approach. Although it proved useful in our case, the cost-benefit ratio of this MLPA-alternative tool must be carefully evaluated considering that both the size of the panel and the non-uniformity of the NGS coverage could lead to a high false-positive detection rate, potentially requiring a great effort for confirmatory analyses. Conversely, if the use of MLPA is considered more favorable, it is necessary to consider the increase in costs and time, especially when many genes require this analysis type.

Of note, despite the known tendency of Ion Torrent technologies to generate false-positive calls represented by the high insertion and deletion (indel) error rate (Quail et al., 2012), we revealed their presence only in the OI cohort and limited to *COL1A1* and *COL1A2*, due to the homopolymer regions typical of collagen genes. Even if the occurrence of false-positive calls does not impact NGS diagnostic reliability, it must be considered as an additional factor influencing the total costs when using the Ion Torrent technology in the presence of specific gene structures. Although for our study the cost impact of false-positive calls—limited to the OI genetic test—is not relevant (a mean of 0.3 confirmation sequences per patient), in the case of more complex gene panels (e.g., for collagenopathies), a more careful evaluation is required.

In addition to the technical considerations, essential for estimating the NGS cost in different scenarios, managing the genetic screening of rare diseases raises several other issues that can be addressed in the context of an in-depth multidimensional analysis. The first series of reflections concerns a detailed analysis of the economic aspects. Unlike most previous studies that were focused on the consumable costs (Stoddard et al., 2014; Al-Mousa et al., 2016), we provided a cost description related to the NGS workflow from DNA extraction to variant detection considering personnel costs, consumables, and equipment (usage and maintenance). As a strength, we considered a real-world scenario where we included in the cost analysis what was spent (in terms of costs and time), without making assumptions.

Our economic analysis showed that an optimal organization based on NGS allows costs reduction in molecular diagnosis, especially those related to materials, and thus improving the sustainability of the service. This is not obvious if we consider that the low incidence of rare diseases, in addition to the use of very small panels, may not benefit from the known cost savings of the NGS approach with a large number of samples and genes analyzed. The mean costs per patient of our NGS methods are € 314 for MO diagnosis (2 genes and 30 amplicons, 12.78 kb) and € 518 for OI diagnosis (14 genes and 195 amplicons, 110.89 kb), in line with a previous study (Marino et al., 2018). We assessed cost-savings per patient of € 74 and € 765 for MO and OI molecular diagnosis, respectively, when moving from traditional single-gene testing to NGS testing. It should be emphasized that the reported values do not represent the total costs related to the clinical diagnosis which must include some additional efforts that are not included in this study (i.e., cost and time for activities not directly linked to NGS activity, overhead costs, and quality cost), estimated to represent about 30–40% of the total costs (Marino et al., 2018). Furthermore, it should be underlined that our costs are representative of an optimal situation: the diagnostic workflow is mainly characterized by automated procedures that reduce personnel costs; the diseases evaluated are characterized by a large percentage of affected patients carrying a pathogenic variant, thus reducing the number of patients requiring the complemented Sanger sequencing step; the target size is quite small, thus limiting the magnitude of the potential complemented Sanger sequencing step; few genes (i.e., *EXT1, EXT2, COL1A1*, and *COL1A2*) require additional MLPA analysis. All these elements are for the benefit of a reduction in costs, making the NGS procedures advantageous even using very small MO genetic panel in routine clinical diagnosis. On the other side, given the ever-increasing development of the NGS technology, a further reduction in material costs foreseen in the future will increasingly favor the use of NGS even considering the less advantageous clinical diagnostic scenarios.

Focusing on the NGS assay and analyzing the consumables costs, related to the greatest absorption of resources, the mean cost is € 244 (range: € 205–306) for MO diagnosis and € 390 (range: € 322–616) for OI molecular screening, revealing an increase in the delta as the target size (in kB) increases. Since the higher costs are associated with the cases in which a pathological variant is not detected, requiring additional tests to examine all not covered regions, a substantial increase in the mean cost is expected in the event of inappropriate clinical diagnoses or as the panel size increases. Alternatively, in the case of NGS panels with the same target size, diseases characterized by a lower percentage of patients usually carrying a pathogenic variant will have higher diagnostic costs.

Considering the two skeletal diseases explored in this study, a large increase in personnel and material cost savings is observed when increasing the panel size (i.e., the number of genes included in the molecular screening). In line with what was previously described (Marino et al., 2018), consumables represent the highest cost driver of the diagnostic process, while labor costs have the lowest impact; this is particularly accentuated in our case due to the high level of automation of molecular procedures, as well as the limited time spent on data analysis which is supported by a user-friendly application for mapping, alignment, and variant detection of NGS data. Not requiring the development of specific pipelines, our strategy represents the minimum investment to develop NGS analysis in the absence of highly specialized personnel. However, a substantial increase in analysis costs—considering both the analysis and development of bioinformatics tools—must be considered when examining many genes, as well as when performing a larger genome analysis (whole-exome and whole-genome sequencing); it has already been predicted that the main cost associated with NGS will increasingly be associated with data analysis rather than data production (Muir et al., 2016).

One of the main limitations in the rare disease diagnosis, potentially impacting the NGS costs, is linked to the flow of patients which is not comparable to more widespread pathologies or oncological diseases. To better manage this low and variable number of patients, our NGS diagnostic approach is based on Ion Torrent PGM technology which is optimized—compared to other available technologies—for limited workloads while also ensuring flexibility. Considering the comparison between the traditional and the NGS-based diagnostic protocol, the budget analysis carried out compared them directly as if they constituted the entire activity of the Genetic Analysis Center. The full use of the tools and personnel involved would allow for better results (by increasing the volumes of analysis) if the fastest NGS analytical paths were used. This would also affect the overall costs of the Genetic Analysis Center. A more in-depth evaluation of this aspect would require the definition of the case-mix of examinations performed by the Genetic Analysis Center. It should be noted that the orthopedic center currently performs 52 analyses per year for MO and 45 analyses per year for OI; despite the low number of samples, the NGS diagnostic protocol was set up (in terms of the number of samples per run) to optimize this specific patient flow. Our experience demonstrated that the NGS protocol—as technically detailed—guarantees cost savings and compliance with an adequate response time for both diseases, even in the case of the extremely disadvantageous situation of a 2 gene (30 amplicons) NGS panel. However, it is necessary to consider that the Istituto Ortopedico Rizzoli is an Italian and European reference center for the rare diseases described in the study, while many laboratories process a lower number of samples or treat ultra-rare diseases which may have a negative impact on costs and diagnostic efficiency. To overcome this limiting factor and to increasingly optimize the diagnostic services, it is necessary to consider the use of multi-disease NGS panels even if resulting in an inevitable increase in material costs. The same multi-disease solution could be implemented in case of a flow patient reduction to maintain the same analysis times or to speed up the diagnostic process.

Moreover, considering the analysis timing, the switching to an NGS protocol will perform the entire path in a shorter period for both diseases; this is true considering both the wet laboratory activities and data analyses. Due to the use of automated procedures from DNA extraction to NGS activities, relevant for both quality results and efficiency, as well as to the high expected mutation rate (90%), the time-saving attribute was also observed in the small disadvantageous MO panel. In addition to making the center more efficient, saving time in molecular diagnosis is essential from a social point of view in terms of a probable increase in accessibility to genetic analyses, in turn leading to a further improvement in both efficiency and benefits. Even though they are rare diseases, it is crucial to consider the heavy burden affecting these patients. Consequently, the opportunity to improve the level of assistance using an NGS approach, without increasing the resources required, appears desirable and to be promoted. From the citizen’s point of view, the NGS protocol implies significant changes in accessibility to diagnostics and reporting, without any change in costs at one’s own expense.

## Conclusion

The diagnostic protocol with the NGS-Ion Torrent^TM^ equipment represents a convenient way to detect mutations in the context of rare diseases, also in the case of a very small, targeted gene panel and a limited number of patients. Overall, even in the absence of direct clinical benefits due to the lack of treatment consequences, the NGS option for molecular diagnosis in our scenario is beneficial and recommended both from a healthcare perspective and affected patients. Here, we described two NGS diagnostic assays, set up to minimize the per-sample sequencing costs, and adjusted to our specific patients’ flow and laboratory organization. As evidenced, any changes in the workflow or disease features will reflect the variation in the estimated costs and turnaround time. To further improve the diagnostic efficiency and to give rare patients the benefits associated with the use of NGS diagnostic tools even in smaller diagnostic centers, the use of multi-disease panels is suggested, although associated with higher costs. Further studies will be required to estimate NGS costs when considering the diagnostic fields which are not advantageous for a targeted NGS approach or requiring very large gene panels, up to whole exome/genome; an essential step also to determine reimbursement for NGS assays, still not completed. As an added goal, despite representing a single institution experience (with specific patient flow and lab organization), the described results can help in the definition of NGS-test pricing. For this purpose, other costs affecting the entire diagnostic process will have to be considered (i.e., overhead costs, cost of personnel not specifically related to NGS activity, accreditation, and certification costs). Overall, the total costs cannot be purely related to the target gene number, unless limiting the analysis to the gene regions well covered by the test while potentially reducing the diagnostic confidence; indeed, we confirmed how the NGS-based diagnostic activity is extremely sensitive to other variables such as the flow of patients (highly variable, especially in the field of rare diseases), the gene complexity (e.g., presence of homopolymer regions), the mutation type (i.e., germline/somatic, SNVs/indel/CNVs), the laboratory organization (i.e., automation of molecular procedures), and the accuracy of clinical diagnosis.

## Data Availability

The original contributions presented in the study are included in the article/Supplementary Material, further inquiries can be directed to the corresponding author.

## Supplementary Material

Supplementary Figure S1 Details of “Traditional” and “NGS-based” molecular screening for MO disease.

Supplementary Figure S2 Details of “Traditional” and “NGS-based” molecular screening for OI disease.
