# Supplementary material for: Real-World Data and Budget Impact Analysis (BIA): Evaluation of a Targeted Next-Generation Sequencing Diagnostic Approach in Two Orthopedic Rare Diseases
Source: Front Pharmacol. 2022 Jun 6;13:785705. doi: 10.3389/fphar.2022.785705 (PMC9207266; doi:10.3389/fphar.2022.785705)
Supplement: Supplementary file 1 [file DataSheet2.PDF]

Supplementary Table 2A - Number of MO patients and number of samples/genetic fragments analysed at each diagnostic step

MULTIPLE OSTEOCHONDROMAS

Single-gene (Traditional) diagnostic protocol

Activity	Number of processed patients	Number of processed samples
DNA extraction (from blood)	181	181
DNA extraction (from buccal swab)	8	8
EXT1 complete DHPLC pre-screening	200	7000 DHPLC runs (35 per patient)
Sanger sequencing of EXT1 abnormal profiles	200	476 (on average 2.4 per patient)
EXT2 complete DHPLC pre-screening	73	2263 DHPLC runs (31 per patient)
Sanger sequencing of EXT2 abnormal profiles	73	202 (on average 2.8 per patient)
MLPA analysis	34	34

NGS diagnostic protocol

Activity	Number of processed patients	Number of processed samples
DNA extraction (from blood)	181	181
DNA extraction (from buccal swab)	8	8
NGS analysis	200	200
Sanger sequencing	200	219 (on average 1.1 per patient)
Real Time PCR	27	97 (on average 3.6 per patient)
MLPA Analysis	1	1
