# Supplementary material for: Real-World Data and Budget Impact Analysis (BIA): Evaluation of a Targeted Next-Generation Sequencing Diagnostic Approach in Two Orthopedic Rare Diseases
Source: Front Pharmacol. 2022 Jun 6;13:785705. doi: 10.3389/fphar.2022.785705 (PMC9207266; doi:10.3389/fphar.2022.785705)
Supplement: Supplementary file 2 [file Table1.pdf]

Supplementary Table 1 - Input data, personnel (cost per hour), and diagnostic devices (acquisition costs)

Healthcare professional	Cost per hour	Source
Technician	€ 20	*Data processing of the Annual Account Report of the Ministry of Economy and Finance (MEF), State General Accounting Department*
Biologist/bioinformatician	€ 41	*Data processing of the Annual Account Report of the Ministry of Economy and Finance (MEF), State General Accounting Department*

Diagnostic devices (MO)	Acquisition cost	Depreciation charge	Resource utilization (%)	Cost to be charged	Activity	Diagnostic test
WAVE System Model 3500HT (Transgenomic)	€ 58,300.0	€ 11,660.0	24%	€ 2,798.4	*DHPLC*	*Single-gene*
3500XL Genetic Analyzer (Thermo Fisher Scientific)	€ 248,820.0	€ 49,764.0	3%	€ 1,492.9	*Sequenziamento SANGER*	*Single-gene*
			0,4%	€ 199	*MLPA*	*Single-gene*
			0,7%	€ 348	*Sequenziamento SANGER*	*NGS*
			0,1%	€ 50	*MLPA*	*NGS*
Biomek 3000 (Beckman Coulter)	€ 30,000.0	€ 6,000.0	3%	€ 180	*Sequenziamento SANGER*	*Single-gene*
			0,7%	€ 42	*Sequenziamento SANGER*	*NGS*
Sequence Pilot (JSI Medical Systems)	€ 18,400.0	€ 3,680.0	3%	€ 110	*Sequenziamento SANGER (data analysis)*	*Single-gene*
			0,7%	€ 26	*Sequenziamento SANGER (data analysis)*	*NGS*
Biomek NX (Beckman Coulter)	€ 95,000.0	€ 19,000.0	23%	€ 4,370.0	*DNA extraction*	*Single-gene*
			14%	€ 2,660.0	*DNA extraction*	*NGS*
Ion PGM System (Thermo Fisher Scientific)	€ 50,000.0	€ 10,000.0	9%	€ 900	*NGS*	*NGS*
Ion Chef (Thermo Fisher Scientific)	€ 45,000.0	€ 9,000.0	9%	€ 810	*NGS*	*NGS*
Rotor-Gene Q (QIAGEN)	€ 29,500.0	€ 5,900.0	0,5%	€ 30	*Real Time PCR*	*NGS*
SEQNEXT (JSI Medical Systems)	€ 18,445.0	€ 3,689.0	3%	€ 111	*NGS data analysis*	*NGS*

Diagnostic devices (OI)	Acquisition cost	Depreciation charge	Resource utilization (%)	Cost to be charged	Activity	Diagnostic test
WAVE System Model 3500HT (Transgenomic)	€ 58,300.0	€ 11,660.0	64%	€ 7,462.4	*DHPLC*	*Single-gene*
3500XL Genetic Analyzer (Thermo Fisher Scientific)	€ 248,820.0	€ 49,764.0	12%	€ 5,971.7	*Sequenziamento SANGER*	*Single-gene*
			0,6%	€ 298	*MLPA*	*Single-gene*
			2%	€ 995	*Sequenziamento SANGER*	*NGS*
			0,6%	€ 298	*MLPA*	*NGS*
Biomek 3000 (Beckman Coulter)	€ 30,000.0	€ 6,000.0	12%	€ 720	*Sequenziamento SANGER*	*Single-gene*
			2%	€ 120	*Sequenziamento SANGER*	*NGS*
Sequence Pilot (JSI Medical Systems)	€ 18,400.0	€ 3,680.0	12%	€ 441	*Sequenziamento SANGER (data analysis)*	*Single-gene*
			2%	€ 73	*Sequenziamento SANGER (data analysis)*	*NGS*
Biomek NX (Beckman Coulter)	€ 95,000.0	€ 19,000.0	35%	€ 6,650.0	*DNA extraction*	*Single-gene*
			27%	€ 5,130.0	*DNA extraction*	*NGS*
Ion PGM System (Thermo Fisher Scientific)	€ 50,000.0	€ 10,000.0	9%	€ 900	*NGS*	*NGS*
Ion Chef (Thermo Fisher Scientific)	€ 45,000.0	€ 9,000.0	9%	€ 810	*NGS*	*NGS*
SEQNEXT (JSI Medical Systems)	€ 18,445.0	€ 3,689.0	15%	€ 553	*NGS data analysis*	*NGS*
