# Supplementary figures and images for: Real-World Data and Budget Impact Analysis (BIA): Evaluation of a Targeted Next-Generation Sequencing Diagnostic Approach in Two Orthopedic Rare Diseases
Source: Front Pharmacol. 2022 Jun 6;13:785705. doi: 10.3389/fphar.2022.785705 (PMC9207266; doi:10.3389/fphar.2022.785705)

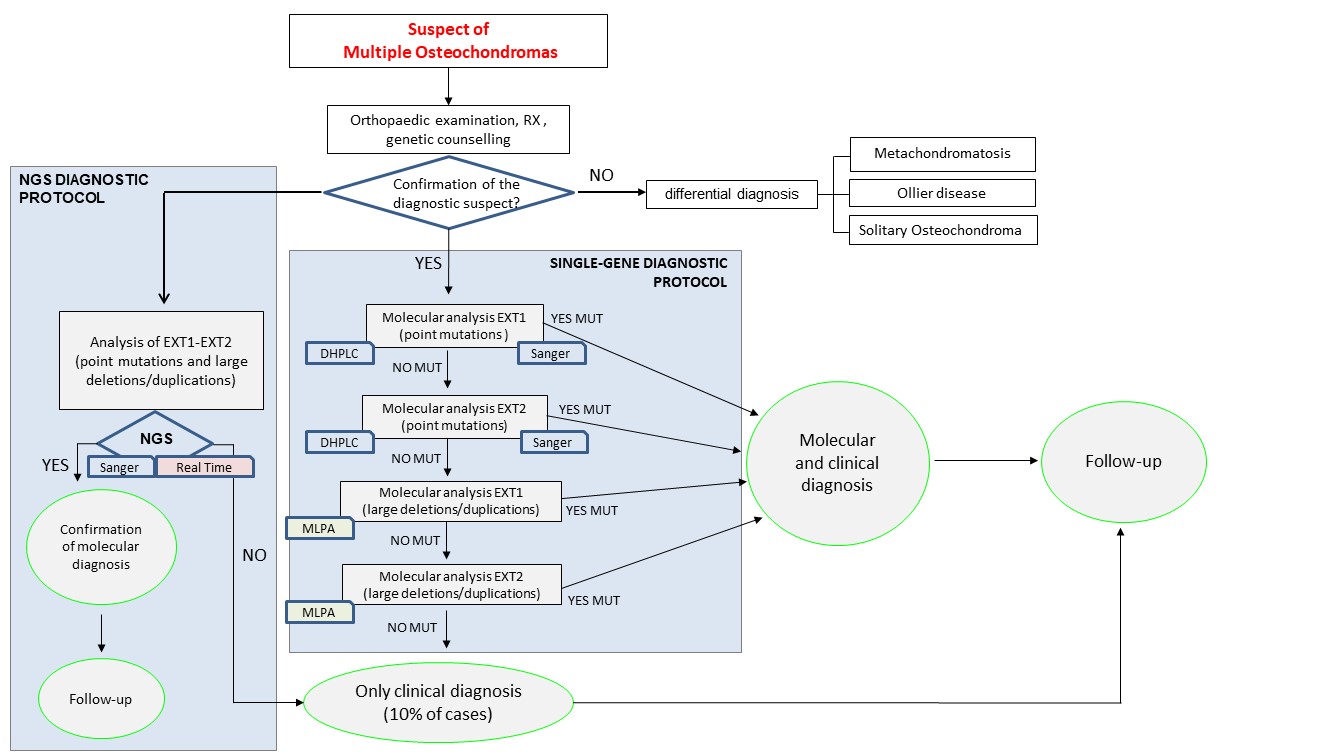

Supplement: Supplementary file 3 [file Image1.JPEG]

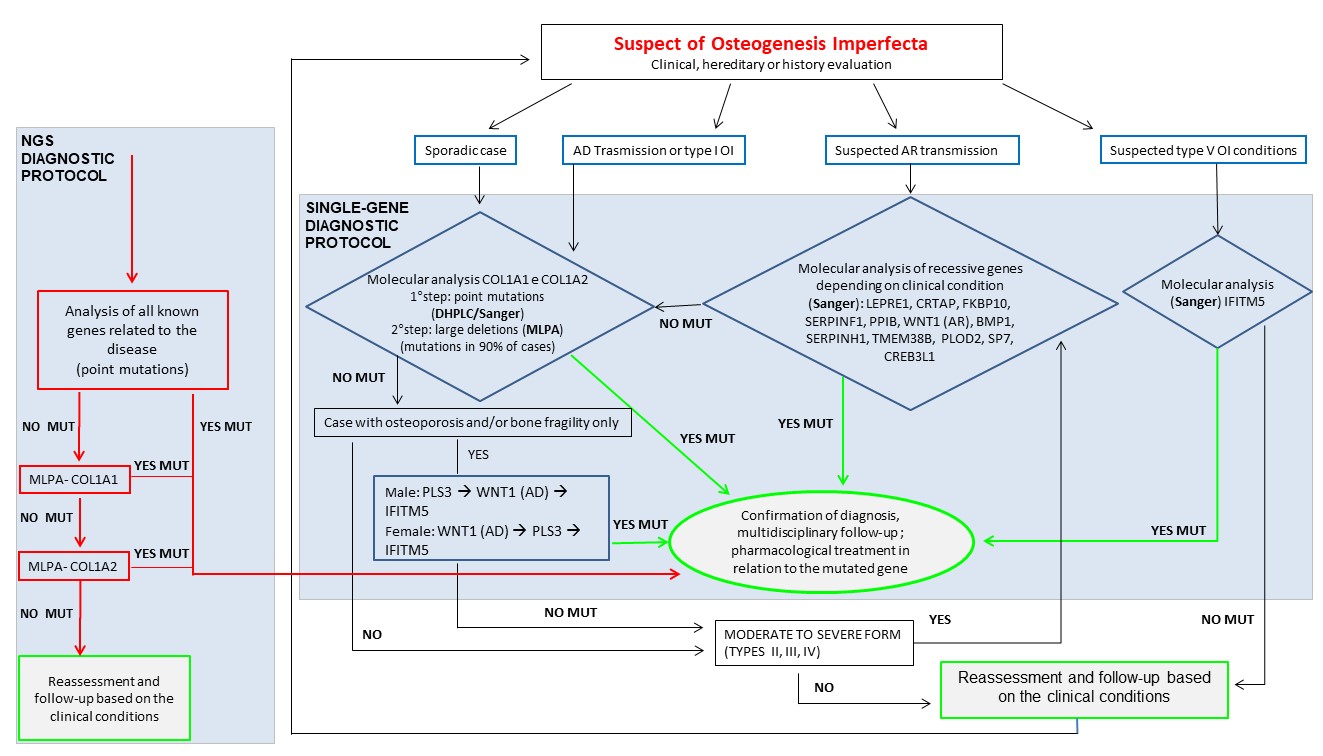

Supplement: Supplementary file 4 [file Image2.JPEG]
