# Supplementary material for: Real-World Data and Budget Impact Analysis (BIA): Evaluation of a Targeted Next-Generation Sequencing Diagnostic Approach in Two Orthopedic Rare Diseases
Source: Front Pharmacol. 2022 Jun 6;13:785705. doi: 10.3389/fphar.2022.785705 (PMC9207266; doi:10.3389/fphar.2022.785705)
Supplement: Supplementary file 5 [file DataSheet3.PDF]

Supplementary Table 2B - Number of OI patients and number of samples/genetic fragments analysed at each diagnostic step

OSTEOGENESIS IMPERFECTA

Single-gene (Traditional) diagnostic protocol

Activity	Number of processed patients	Number of processed samples
DNA extraction (from blood)	187	298 (1.6 extraction per patient)
DNA extraction (from buccal swab)	2	2
COL1A1 complete DHPLC pre-screening	190	15390 DHPLC runs (81 per patient)
Sanger sequencing of COL1A1 abnormal profiles	190	1842 (on average 9.7 per patient)
COL1A2 complete DHPLC pre-screening	128	14080 DHPLC runs (110 per patient)
Sanger sequencing of COL1A2 abnormal profiles	128	789 (on average 6.2 per patient)
COL1A1 MLPA analysis	61	61
COL1A2 MLPA analysis	60	60
IFITM5 sequencing analysis (2 Sanger fragments)	5	10 (on average 2 per patient)
CRTAP sequencing analysis (8 Sanger fragments)	26	218 (on average 8.4 per patient)
LEPRE1 sequencing analysis (16 Sanger fragments)	28	436 (on average 15.6 per patient)
PPIB sequencing analysis (5 Sanger fragments)	22	111 (on average 5 per patient)
SERPINF1 sequencing analysis (6 Sanger fragments)	21	194 (on average 9.2 per patient)
WNT1 sequencing analysis (5 Sanger fragments)	18	92 (on average 5.1 per patient)
TMEM38B sequencing analysis (6 Sanger fragments)	1	6 (on average 6 per patient)
BMP1 sequencing analysis (20 Sanger fragments)	1	20 (on average 20 per patient)
SERPINH1 sequencing analysis (5 Sanger fragments)	14	63 (on average 4.5 per patient)
FKBP10 sequencing analysis (10 Sanger fragments)	20	195 (on average 9.75 per patient)
SP7 sequencing analysis (6 Sanger fragments)	13	78 (on average 6 per patient)
PLS3 sequencing analysis (15 Sanger fragments)	2	32 (on average 16 per patient)

NGS diagnostic protocol

Activity	Number of processed patients	Number of processed samples
DNA extraction (from blood)	187	187
DNA extraction (from buccal swab)	2	2
NGS analysis	199	199
Sanger sequencing	199	555 (on average 2.8 per patient)
COL1A1 MLPA analysis	51	51
COL1A2 MLPA analysis	50	50
