# Supplementary material for: Real-World Data and Budget Impact Analysis (BIA): Evaluation of a Targeted Next-Generation Sequencing Diagnostic Approach in Two Orthopedic Rare Diseases
Source: Front Pharmacol. 2022 Jun 6;13:785705. doi: 10.3389/fphar.2022.785705 (PMC9207266; doi:10.3389/fphar.2022.785705)
Supplement: Supplementary file 6 [file DataSheet1.PDF]

## Supplementary Table 1 - Input data, personnel (cost per hour), and diagnostic devices (acquisition costs)

**Healthcare professional**

| | Cost per hour | Source |
|---|---|---|
| Technician | € 20 | *Data processing of the Annual Account Report of the Ministry of Economy and Finance (MEF), State General Accounting Department* |
| Biologisti/bioinformatician | € 41 | *Data processing of the Annual Account Report of the Ministry of Economy and Finance (MEF), State General Accounting Department* |

**Diagnostic devices (MO)**

| | Acquisition cost | Depreciation charge | Resource utilization (%) | Cost to be charged | Activity | Diagnostic test |
|---|---|---|---|---|---|---|
| WAVE System Model 3500HT (Transgenomic) | € 58,300.0 | € 11,660.0 | 24% | € 2,798.4 | *DHPLC* | *Single-gene* |
| 3500XL Genetic Analyzer (Thermo Fisher Scientific) | € 248,820.0 | € 49,764.0 | 3% | € 1,492.9 | *Sequenziamento SANGER* | *Single-gene* |
| | | | 0,4% | € 199 | *MLPA* | *Single-gene* |
| | | | 0,7% | € 348 | *Sequenziamento SANGER* | *NGS* |
| | | | 0,1% | € 50 | *MLPA* | *NGS* |
| Biomek 3000 (Beckman Coulter) | € 30,000.0 | € 6,000.0 | 3% | € 180 | *Sequenziamento SANGER* | *Single-gene* |
| | | | 0,7% | € 42 | *Sequenziamento SANGER* | *NGS* |
| Sequence Pilot (JSI Medical Systems) | € 18,400.0 | € 3,680.0 | 3% | € 110 | *Sequenziamento SANGER (data analysis)* | *Single-gene* |
| | | | 0,7% | € 26 | *Sequenziamento SANGER (data analysis)* | *NGS* |
| Biomek NX (Beckman Coulter) | € 95,000.0 | € 19,000.0 | 23% | € 4,370.0 | *Estrazione DNA* | *Single-gene* |
| | | | 14% | € 2,660.0 | *Estrazione DNA* | *NGS* |
| Ion PGM System (Thermo Fisher Scientific) | € 50,000.0 | € 10,000.0 | 9% | € 900 | *NGS* | *NGS* |
| Ion Chef (Thermo Fisher Scientific) | € 45,000.0 | € 9,000.0 | 9% | € 810 | *NGS* | *NGS* |
| Rotor-Gene Q (QIAGEN) | € 29,500.0 | € 5,900.0 | 0,5% | € 30 | *Real Time PCR* | *NGS* |
| SEQNEXT (JSI Medical Systems) | € 18,445.0 | € 3,689.0 | 3% | € 111 | *NGS data analysis* | *NGS* |

**Diagnostic devices (OI)**

| | Acquisition cost | Depreciation charge | Resource utilization (%) | Cost to be charged | Activity | Diagnostic test |
|---|---|---|---|---|---|---|
| WAVE System Model 3500HT (Transgenomic) | € 58,300.0 | € 11,660.0 | 64% | € 7,462.4 | *DHPLC* | *Single-gene* |
| 3500XL Genetic Analyzer (Thermo Fisher Scientific) | € 248,820.0 | € 49,764.0 | 12% | € 5,971.7 | *Sequenziamento SANGER* | *Single-gene* |
| | | | 0,6% | € 298 | *MLPA* | *Single-gene* |
| | | | 2% | € 995 | *Sequenziamento SANGER* | *NGS* |
| | | | 0,6% | € 298 | *MLPA* | *NGS* |
| Biomek 3000 (Beckman Coulter) | € 30,000.0 | € 6,000.0 | 12% | € 720 | *Sequenziamento SANGER* | *Single-gene* |
| | | | 2% | € 120 | *Sequenziamento SANGER* | *NGS* |
| Sequence Pilot (JSI Medical Systems) | € 18,400.0 | € 3,680.0 | 12% | € 441 | *Sequenziamento SANGER (data analysis)* | *Single-gene* |
| | | | 2% | € 73 | *Sequenziamento SANGER (data analysis)* | *NGS* |
| Biomek NX (Beckman Coulter) | € 95,000.0 | € 19,000.0 | 35% | € 6,650.0 | *Estrazione DNA* | *Single-gene* |
| | | | 27% | € 5,130.0 | *Estrazione DNA* | *NGS* |
| Ion PGM System (Thermo Fisher Scientific) | € 50,000.0 | € 10,000.0 | 9% | € 900 | *NGS* | *NGS* |
| Ion Chef (Thermo Fisher Scientific) | € 45,000.0 | € 9,000.0 | 9% | € 810 | *NGS* | *NGS* |
| SEQNEXT (JSI Medical Systems) | € 18,445.0 | € 3,689.0 | 15% | € 553 | *NGS data analysis* | *NGS* |
